# SUMO-Activating Enzyme Subunit 1 Is Associated with Poor Prognosis, Tumor Progression, and Radio-Resistance in Colorectal Cancer

**DOI:** 10.3390/cimb45100506

**Published:** 2023-09-30

**Authors:** Yueh-Jung Wu, Siang-Ting Huang, Ya-Hui Chang, Shih-Yi Lin, Weng-Ling Lin, Ying-Jung Chen, Shang-Tao Chien

**Affiliations:** 1 Division of Colorectal Surgery, Kaohsiung Armed Forces General Hospital, Kaohsiung 802, Taiwan; allenyjw@gmail.com; 2 Cancer Center, Kaohsiung Armed Forces General Hospital, Kaohsiung 802, Taiwan; ting1020622@gmail.com (S.-T.H.); next660918@gmail.com (Y.-H.C.); 3 Department of Pathology, Kaohsiung Armed Forces General Hospital, Kaohsiung 802, Taiwanwinnielin6204@gmail.com (W.-L.L.); 4 Department of Fragrance and Cosmetic Science, Kaohsiung Medical University, Kaohsiung 807, Taiwan; yjchen@kmu.edu.tw; 5 Department of Nursing, Fooyin University, Kaohsiung 831, Taiwan

**Keywords:** SAE1, chemoradiotherapy, colorectal carcinoma, prognosis, biomarker

## Abstract

Concurrent chemoradiotherapy is an effective treatment option for patients with low-grade colorectal cancer (CRC) in the local disease stage. At present, the principle of the Taiwan Medical Center is to treat CRC patients with combination radiotherapy and chemotherapy (high-dose 5-FU) for a period of about five weeks prior to surgery. Radical resection of the tumor is performed at least six to eight weeks after concurrent chemoradiotherapy (CCRT). However, this approach fails to produce the desired therapeutic effect in approximately 20% to 30% of patients, and such patients are unnecessarily exposed to the risks of radiation and drug toxicity posed by this therapy. Therefore, it is crucial to explore new biomarkers to predict the prognosis of CRC. SUMO-activating enzyme subunit 1 (SAE1) plays an important role in SUMOylation, a post-translational modification involved in cellular functions, such as cell proliferation, cell cycle, and apoptosis. In our study, to explore the clinical–pathological role of SAE1 protein in CRC, we evaluated the clinical data and paraffin sections from CRC patients. The expression of SAE1 was evaluated using immunohistochemical analysis, and clinical parameters were analyzed using chi-square and Kaplan–Meier survival tests. The results of in vitro proliferation and radiosensitive assays were compared between control groups and SAE1 siRNA groups. Western blotting was also used to detect the expressions of the SAE1, PARP, cyclin D1, p-NF-κB, and NF-κB proteins. Flow cytometry and colony formation assays were used to detect the effect of SAE-1 on radiosensitivity. In vivo, we detected the growth curve in a mouse xenograft model. The results showed that SAE-1 was revealed to be an independent prognostic biomarker of CRC. SAE1 knockdown inhibited CRC proliferation in vitro and in vivo, and led to the cleavage of PARP, downregulation of cyclin D1 protein expression, and downregulation of p-NF-κB/NF-κB. Additionally, SAE1 knockdown promoted radiosensitivity in CRC cells. Therefore, it was inferred that SAE1 may be used as a potential therapeutic target in CRC treatment.

## 1. Introduction

Colorectal cancer (CRC) is one of the most common gastrointestinal cancers in the world. Approximately 9 million CRC cases and 900,000 CRC-related deaths were reported globally in 2020 [1]. The standard treatment of CRC comprises surgery combined with chemoradiotherapy (CCRT) [2,3]. Surgery is often the first line of treatment for colorectal cancer. The surgeon may remove the tumor along with nearby lymph nodes, or in more advanced cases, the entire colon or rectum. In some cases, a temporary or permanent colostomy may be needed to divert waste out of the body. Radiation therapy uses high-energy radiation to kill cancer cells or shrink tumors [2,3]. It is sometimes used before surgery to shrink the tumor or after surgery to kill any remaining cancer cells. Chemotherapy uses drugs to kill cancer cells and is often used in combination with surgery or radiation therapy [2,3]. In addition to these treatments, there are also targeted therapies that specifically target the cancer cells, immunotherapy that boost the body’s immune system to fight the cancer cells, and palliative care that aims to improve the quality of life for people with advanced or terminal colorectal cancer. Although surgery with concurrent CCRT can improve survival time, CRC recurrence continues to be a major factor affecting patient prognosis [4,5,6,7]. Furthermore, CCRT fails to improve prognosis in 20–30% of CRC patients [8,9,10]. Therefore, biomarkers capable of predicting prognosis and recurrence are imperative to the success of adjuvant therapy in CRC.

SUMOylation, a type of post-translational modification, is a small ubiquitin-like modifier (SUMO)-dependent biological modification process that regulates several biological functions, including tumor development in many cancers [11,12]. The enzymatic cascade of SUMOylation involves three steps: heterodimer E1 enzyme (SAE1 and SAE2/UBA2)-involved activation, E2 enzyme Ubc9-regulated conjugation, and substrate modification following the coaction of E2 and E3 protein ligases [11,13]. Previous studies have reported SAE1 as a prognostic biomarker in hepatocellular carcinoma (HCC) [14], triple-negative breast cancer [15], and glioma [16]. However, little is known about the association between the protein expression of SAE1 and clinicopathological parameters of malignancies, such as CRC. Therefore, the current study aimed to examine the expression of SAE1 in CRC using immunohistochemical (IHC) staining to explore its clinical significance in CRC patients. In addition, using SAE1 siRNA confirmed the correlation between SAE1 and tumor progression.

## 2. Materials and Methods

### 2.1. Patients

A total of 200 CRC patients receiving pre-operative care that had total meso-rectal resection for tumor excision and lymph node dissection at Kaohsiung Armed Forces General Hospital were initially selected for this study. However, 19 cases were excluded due to lack of post-biopsy follow-up data, and 20 were excluded because of low-quality pathological results or poor IHC staining. Therefore, 161 patients were included in the final analysis. This study was approved by the Institutional Review Board of the Kaohsiung Armed Forces General Hospital (KAFGH 107-038).

### 2.2. IHC Staining

Formalin-fixed, paraffin-embedded tissue blocks prepared from CRC biopsy specimens of the included participants were used to obtain 3 µm thick tissue sections for IHC staining. The sections were deparaffinized with xylene, rehydrated with alcohol, and subjected to antigen retrieval at 121 °C for 10 min in Target Retrieval Solution at a pH of 6.0 (DAKO, Santa clara, USA; S2369). Hydrogen peroxide (3%) was used to block endogenous peroxidase activity for 5 min at room temperature. Thereafter, the slides were incubated with 1:200 dilution of anti-SAE1 polyclonal antibody (proteintech, Rosemont, USA; 10229-1-AP) for 1 h at room temperature, and subsequently treated with secondary antibodies conjugated with horseradish peroxidase for 30 min at room temperature. Finally, the slides were incubated in 3,3-diaminobenzidine (Dako; K5007) for 5 min and in Mayer’s hematoxylin counterstain for 2 min. Lastly, the IHC staining scores of SAE1 were calculated and used to classify the samples into two intensity categories: low-level expression and high-level expression. Scores, which represented the proportions of positively stained tumor cells, were determined as follows: 0, <10% positive tumor cells; 1, 10–40% positive cells; 2, 40–70% positive cells; and 3, >70% positive cells. The staining intensity was classified as 0, no staining; 1, weak staining; 2, moderate staining; or 3, strong staining. The staining index (SI) was calculated by multiplying the intensity and percentage of positive tumor cells in each sample to yield possible scores of 0, 1, 2, 3, 4, 6, and 9. We set a total score of 4 as a cut-off; in other words, ≥4 was considered high SAE1 expression, and <3 was considered low expression.

### 2.3. Cell Culture

SW620 and HCT116 cells were purchased from the Bioresource Collection and Research Center (Hsinchu, Taiwan) and cultured in Dulbecco’s Modified Eagle Medium (DMEM) (Gibco; 12800-017, Waltham, MA, USA) with 10% fetal bovine serum (FBS) at 37 °C in an atmosphere of 5% CO_2_.

### 2.4. Transfection

CRC cells were transfected with 5 μM SAE1 siRNA or non-target sense RNA (negative control) using the DharmaFECT Transfection Reagents (DharmaconTM, Massachusetts, USA), according to the manufacturer’s instructions. The cells were divided into four groups in this study: control, negative control, si-SAE1#1, and si-SAE1#2. The SAE1 siRNA #1 sequence was GUUCCGUACAGAUAAAGGA, and the SAE1 siRNA #2 sequence was UCCCAGUUCUGAUACAUAU.

### 2.5. Cell Viability

In a 24-well plate, 3 × 10^4^ cells were seeded in 0.5 mL of medium in each well. Cell viability was detected by performing an MTT assay at 24 h, 48 h, and 72 h after transfection with 5 μM SAE1 siRNA or non-target sense RNA (*n* = 6).

### 2.6. Western Blot Analysis

A total of 3 × 10^5^ cells were seeded in a 6-well plate and transfected with 5 μM SAE1 siRNA or nontarget sense RNA. All cell groups were lysed using the RIPA buffer. Subsequently, 50 μg protein of each sample was loaded into 10–12% SDS-PAGE wells and electrophoresis was conducted at 50 V for 4 h and then transferred from the gel to the PVDF membrane at 200 mA for 2 h. After 1 h incubation with a blocking buffer, the membranes were incubated with primary antibodies (anti-SAE1 polyclonal antibody (1:500; proteintech; 10229-1-AP), anti-cleaved caspase-3 monoclonal antibody (1:500; Cell signaling, MA, USA; #9664), anti-β-actin monoclonal antibody (1:20,000; Sigma; St Louis, MI, USA; A5541), anti-cyclin D1 monoclonal antibody (1:500; proteintech; 60186-1-lg), anti-PARP polyclonal antibody (1:500; Cell Signaling; #9542), anti-NF-κB monoclonal antibody (1:500; Cell Signaling; #6956), and anti-p-NF-κB monoclonal antibody (1:500; Cell Signaling; #3033)) overnight and with secondary antibodies (Goat anti-Rabbit (1:5000; Millipore, MA, USA; AP132P) and Goat anti-Mouse (1:5000; Millipore; AP124P)) for 90 min. Thereafter, an ECL substrate solution (Western Lightning, MA, USA; 205-14621) was used to detect specific bands with a Minichemi chemiluminescence imaging instrument (ThermoFisher Scientific Inc, MA, USA) (*n* = 4).

### 2.7. Flow Cytometry

A total of 3 × 10^5^ cells were seeded in a 6-well plate and transfected with 5 μM SAE1 siRNA or non-target sense RNA. After 24 h, the supernatants were collected and the adhesion cells were washed with PBS and incubated with TrypLE^TM^ (Gibco; 12605-028) for 5 min. The supernatants and the cells were collected through centrifuge (1500 rpm, 5 min). The apoptosis assay was detected by using the Muse^®^ Annexin V & Dead Cell Kit (Millipore; MCH100105, Burlington, MA, USA). Data were evaluated using the Muse™ cell analyzer (*n* = 4).

### 2.8. Colony Formation

After transfection with 5 μM SAE1 siRNA or non-target sense RNA, 100, 200, 400, 800, and 1000 cells were individually seeded in a 6-well plate and assessed after successive irradiation at doses of 0, 1, 2, 4, and 8 Gy. On the tenth day, the cells were fixed using methanol for 10 min and stained with Coomassie brilliant blue. The plating efficiency (PE) was determined in the control cells as the number of counted colonies/seeded cells. The survival fraction (SF) was calculated as the number of colonies formed after treatment/(cells seeded × PE) (*n* = 4).

### 2.9. Animal Model

There experimental animals were divided into two groups: control and SAE1 knockdown groups (*n* = 12). HCT116 cells (1 × 10^6^ cells in 100 μL PBS) were subcutaneously injected into NU/NU nude mice (NxGen BioSciences). Seven days after injection with HCT116 cells, 10 μL PBS including 5 μM SAE1 siRNA (SAE1 knock-down group) or PBS (control group) was intratumorally injected every three days. Tumor volume (mm^3^) was measured every week and calculated as (length × width^2^)/2 on days 7, 14, 21, 28, 35, and 42. The animal experiments were reviewed and approved by the Institutional Animal Care and Use Committee of Kaohsiung Medical University (IACUC Approval No:110107).

### 2.10. Data Analysis

SPSS ver. 19.0 (Chicago, IL, USA) was used for the statistical analyses of this study. A chi-squared test was performed to determine whether there was a correlation between SAE1 protein expression and specific clinicopathological parameters (sex, age, tumor size, T stage, N stage, M stage, pathologic stage, recurrent, vascular invasion, and perineural invasion) of CRC. Survival rates were analyzed using the Kaplan–Meier method with a log-rank test. Multivariate Cox regression analysis was used to verify the independent effects of each variable involved in the study. The results of Western blot analysis were interpreted through LaneImage 1D gel analysis. A *p* value of <0.05 was considered to be statistically significant.

## 3. Results

### 3.1. Correlation between SAE1 Expression and Clinical Parameters

**Figure 1:**
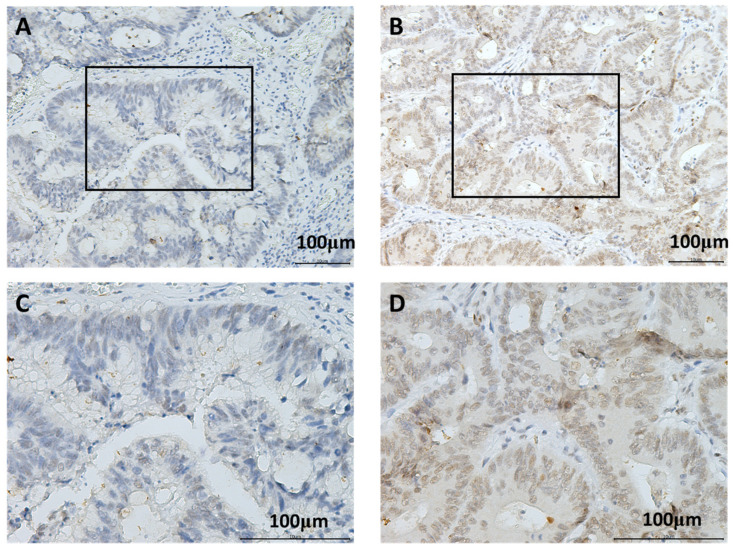
Representative results of immunohistochemical staining for SAE1 using samples obtained from patients with different immunohistochemical staining scores: (**A**) CRC with low level of SAE1 expression, 200×; (**B**) CRC with high level of SAE1 expression, 200×; (**C**) CRC with low level of SAE1 expression, 400×; (**D**) CRC with high level of SAE1 expression, 400×. The black squares represent the position of C and D on the specimen.

The study cohort consisted of 161 patients with CRC (61 females and 100 males), including 37 patients aged ≥60 years and 124 patients aged <60 years. The tumors of 60 patients were ≥5 cm, while those of the remaining 101 were <5 cm in size. The tumors were classified as T1/2 in 56 cases and T3/4 in 105 cases. The tumors of 94 patients were classified as N0 and 67 were classified as N1/N2; 120 patients had M0 tumors, while 41 had M1 tumors. A total of 83 patients were classified as having stage I/II, while 78 were classified as having stage III/IV malignancy. Furthermore, 60 patients were diagnosed with recurrent disease, 78 with vascular invasion, and 31 with perineural invasion (Table 1). As shown in Figure 1, SAE1 was expressed in the nucleus. SAE1 staining was performed to identify correlations between SAE1 expression and the clinical parameters of patients with CRC. The results revealed that SAE1 protein expression was not significantly correlated with sex (*p* = 0.414), age (*p* = 0.187), tumor size (*p* = 0.191), T stage (*p* = 1), N stage (*p* = 0.053), pathological stage (*p* = 0.081), tumor recurrence (*p* = 0.327), vascular invasion (*p* = 0.268), or perineural invasion (*p* = 0.423). However, SAE1 protein expression was significantly correlated with M stage (*p* = 0.006) (Table 1).

### 3.2. Survival Analysis

**Figure 2:**
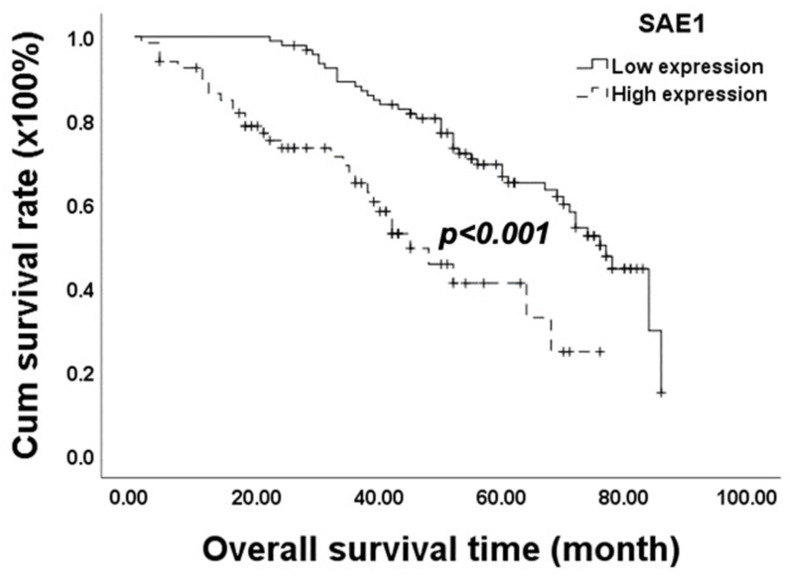
Analysis of SAE1 protein expression using Kaplan−Meier analysis. A cohort of 161 CRC patients was evaluated. Significance was determined using the log-rank test.

The results of the Kaplan–Meier analysis and log-rank test revealed that high SAE1 expression was significantly correlated with poor overall survival (OS) (*p* < 0.001; Figure 2). Regarding OS, the results of the univariate analysis showed that CRC patients with low-level SAE1 expression lived significantly longer than those with high SAE1 expression (HR = 0.340; 95% CI = 0.205–0.562; *p* < 0.001). In addition, the T stage (HR = 0.543; 95% CI = 0.313–0.941; *p* = 0.029), N stage (HR = 0.323; 95% CI = 0.200–0.521; *p* < 0.001), M stage (HR = 0.100; 95% CI = 0.059–0.170; *p* < 0.001), pathological stage (HR = 0.215; 95% CI = 0.128–0.361; *p* < 0.001), recurrence (HR = 0.567; 95% CI = 0.355–0.907; *p* = 0.018), vascular invasion (HR = 0.339; 95% CI = 0.207–0.554; *p* < 0.001), and perineural invasion (HR = 0.564; 95% CI = 0.336–0.947; *p* = 0.031) of the patient were found to be significantly correlated with OS.

However, results of the multivariate analysis revealed a significant correlation between OS and M stage (HR = 0.170; 95% CI = 0.085–0.339; *p* < 0.001) and SAE1 expression (HR = 0.383; 95% CI = 0.223–0.655; *p* < 0.001) (Table 2).

### 3.3. SAE1 Protein Expression with SAE1 siRNA in CRC Cells

**Figure 3:**
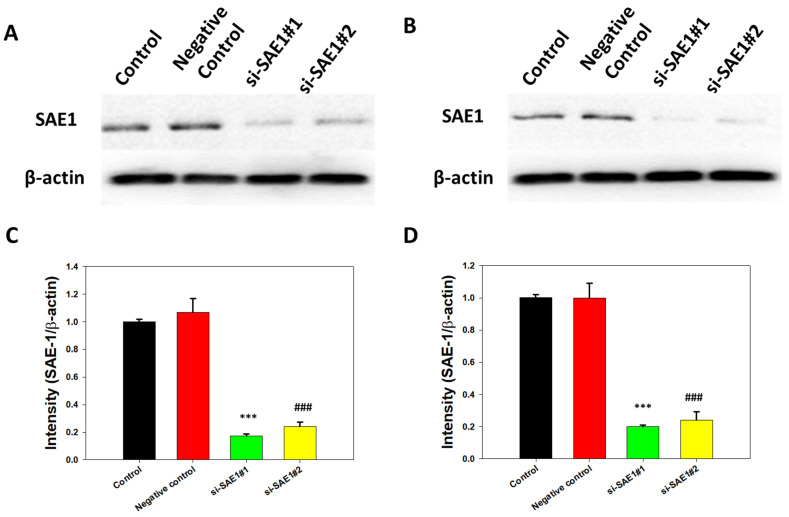
SAE1 expression in control, negative control, and SAE1 siRNA groups in SW620 and HCT116 cells. Western blotting for SAE1 expression in SW620 (**A**) and HCT116 (**B**) cells; relative SAE1 protein expression for SAE1 expression in SW620 (**C**) and HCT116 (**D**) cells. *** *p* < 0.001 compared between si-SAE1#1 group and control group. ### *p* < 0.001 compared between si-SAE1#2 group and control group.

After 48 h transfection with SAE1 siRNA (si-SAE1 #1 and #2) or nonsense siRNA (negative control group), SAE1 protein expression levels were compared between the negative control and siRNA groups using Western blot analysis. The results revealed that SAE1 knockdown with si-SAE1 #1 and #2 reduced SAE1 protein expression in SW620 cells (Figure 3A,C). Similarly, SAE1 protein expression reduction was also seen in HCT116 cells following SAE1 knockdown with si-SAE1 #1 and #2 (Figure 3B,D). These findings suggested that SAE1 knockdown successfully downregulated SAE1 protein expression, with significant differences between the positive and negative control groups.

### 3.4. Silencing of SAE1 Inhibited CRC Cell Proliferation

**Figure 4:**
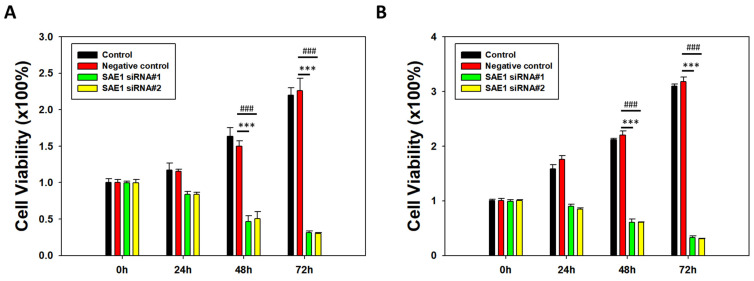
MTT assay of CRC cells treated with SAE1 siRNA. Bar chart of CRC cells cultured with SAE1 siRNA for 0 h, 24 h, 48 h, and 72 h in 24-well plates in (**A**) SW620 and (**B**) HCT116 cells. *** *p* < 0.001 compared between si-SAE1#1 group and control group. ### *p* < 0.001 compared between si-SAE1#2 group and control group.

An MTT assay was used to evaluate the effects of SAE1 knockdown on the proliferation of SW620 and HCT116 cells, by assessing cell viability after transfection with siRNA for 24, 48, or 72 h. In SW620 cells, there was decreased cell viability in the si-SAE1 #1 and #2 groups compared to the control group after 24 and 72 h of incubation (Figure 4A). In HCT116 cells, there was decreased cell viability in the si-SAE1 #1 group compared with the control group after 24 h of transfection, and in the si-SAE1 #1 and #2 groups after 72 h of transfection (Figure 4B). Furthermore, there was no significant difference in the viability of SW620 and HCT116 cells between the positive and negative control groups. Overall, these findings indicate a correlation between SAE1 knockdown and decreased cell viability in CRC.

### 3.5. SAE1 Silencing Regulated PARP, Cyclin D1, p-NF-κB, and NF-κB Protein Expression

**Figure 5:**
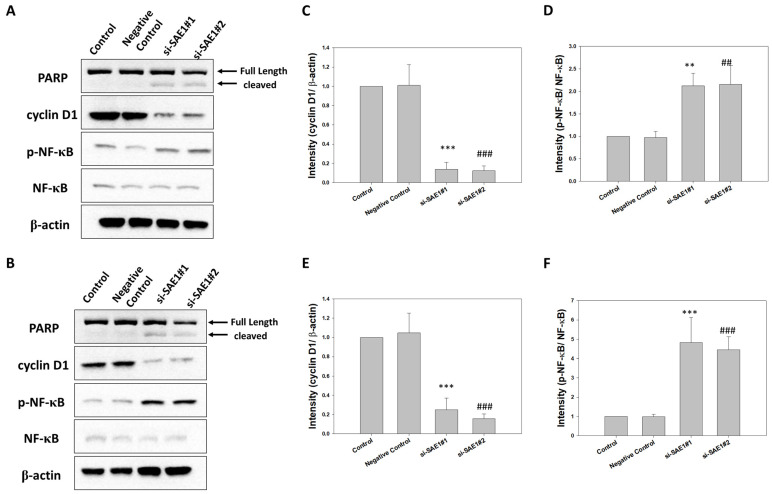
Expression levels of PARP, cyclin D1, p-NF-κB, and NF-κB proteins in control, negative control, and SAE1 siRNA-treated cells. Western blot analysis to assess PARP, cyclin D1, p-NF-κB, and NF-κB expression in (**A**) HCT116 cells and (**B**) SW620 cells. Relative protein expression levels of (**C**) cyclin D1 and (**D**) p-NF-κB/NF-κB in HCT116 cells. Relative protein expression levels of (**E**) cyclin D1 and (**F**) p-NF-κB/NF-κB in SW620 cells. ** *p* < 0.01 and *** *p* < 0.001 compared between si-SAE1#1 group and control group. ## *p* < 0.01 and ### *p* < 0.001 compared between si-SAE1#2 group and control group.

Western blot analysis was employed to elucidate the functional role of SAE1 in the regulation of protein expressions related to key cellular processes in CRC, including PARP, cyclin D1, p-NF-κB, and NF-κB. This analysis was performed subsequent to the knockdown of SAE1 in HCT116 (Figure 5A) and SW620 (Figure 5B) cells. Notably, PARP serves as an apoptosis marker, while Cyclin D1 is indicative of cell proliferation. Additionally, NF-κB functions as a pivotal transcription factor in the modulation of inflammation. Our findings revealed that upon SAE1 knockdown, a series of significant alterations occurred, including the cleavage of PARP, along with the downregulation of both cyclin D1 and p-NF-κB/NF-κB protein expressions in HCT116 (Figure 5C,D) and SW620 (Figure 5E,F).

### 3.6. SAE1 siRNA Attenuated Growth of CRC in Nude Mice

**Figure 6:**
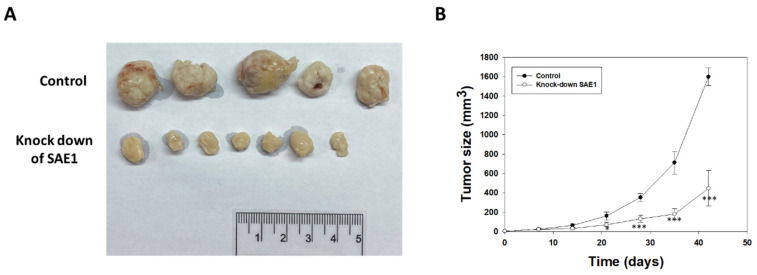
Tumor xenograft growth in nude mice. (**A**) Pictures of gross examination and (**B**) plot tumor growth curves showing inhibitory effect of SAE1 siRNA on human colon carcinoma cell tumor xenograft growth in nude mice. * *p* < 0.05, *** *p* < 0.001 compared to control group.

On the seventh day after injection with HCT116 cells, SAE1 siRNA was intratumorally injected every three days. Tumor size was measured every week and calculated as (length × width^2^)/2 on days 7, 14, 21, 28, 35, and 42 (Figure 6A). Our results showed that treatment with SAE1 siRNA attenuated tumor growth at 21, 28, 35, and 42 days (Figure 6B). Therefore, SAE1 siRNA has theoretical potential to be used as a therapeutic modality in CRC.

### 3.7. SAE1 Mediated Effect of Radiotherapy in CRCs

**Figure 7:**
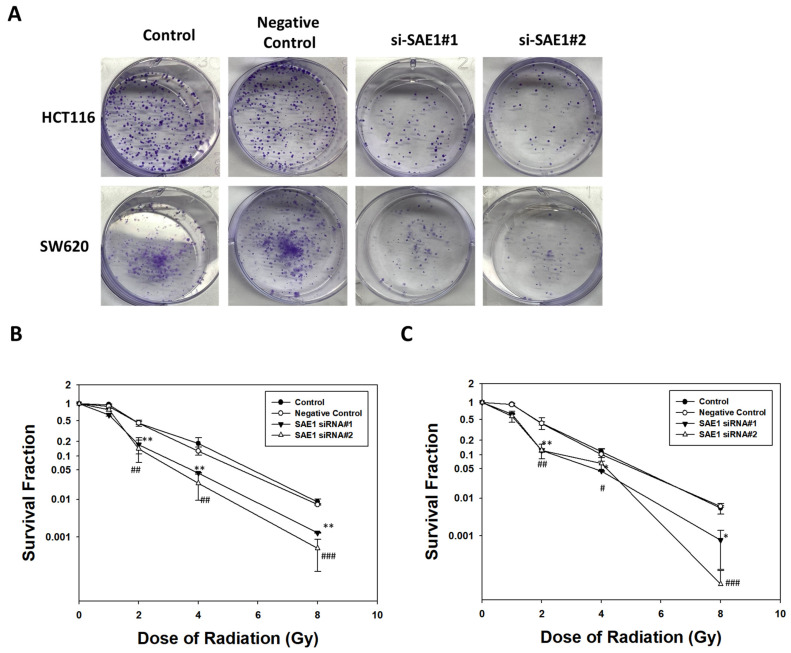
Colony formation after irradiation in control, negative control, and SAE1 siRNA-treated cells. (**A**) Cell clone formation ability detected by colony formation assay after irradiating cells with a dose of 2 Gy on day 10. (**B**) Colony formation assay used to measure colony survival rate 10 days after being exposed to the indicated single doses of irradiation (0, 1, 2, 4, or 8 Gy) in HCT116 cells. (**C**) Colony formation assay used to measure colony survival rate ten days after being exposed to the indicated single doses of irradiation (0, 1, 2, 4, or 8 Gy) in SW620 cells. * *p* < 0.05 and ** *p* < 0.01 compared between si-SAE1#1 group and control group. # *p* < 0.05, ## *p* < 0.01 and ### *p* < 0.001 compared between si-SAE1#2 group and control group.

**Figure 8:**
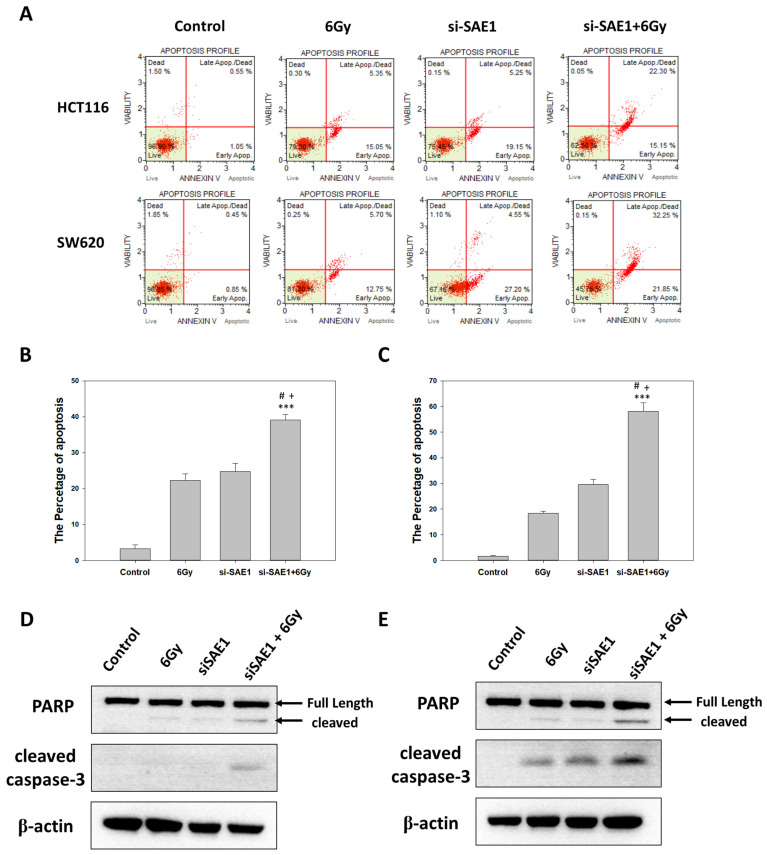
Apoptosis assay in control, 6 Gy, SAE1 siRNA, and SAE siRNA + 6 Gy groups. (**A**) Double stain for apoptosis in control, 6 Gy, SAE1 siRNA, and SAE siRNA + 6 Gy groups using flow cytometry. (**B**) Quantification of apoptosis in HCT116 cells. (**C**) Quantification of apoptosis in SW620 cells. (**D**) Western blot analysis for PARP and cleaved caspase-3 in HCT116 cells. (**E**) Western blot analysis for PARP and cleaved caspase-3 in SW620 cells. *** *p* < 0.001 compared to control group. # *p* < 0.05 compared to 6 Gy group. + *p* < 0.05 compared to si-SAE1 group.

Colony formation and flow cytometry were used to analyze the correlation between SAE1 and radiation. With respect to colony formation, the group with SAE1 siRNA had fewer colony formations than the control group at 2 Gray (Gy) (Figure 7A) and higher sensitivity in survival fraction after radiation in HCT116 (Figure 7B) and SW620 (Figure 7C) cells. Due to the better efficacy of SAE1 siRNA#2 compared to siRNA#1, we chose to utilize SAE1 siRNA#2 for subsequent experiments involving flow cytometry and Western blot analyses. We compared apoptosis between the control group, 6 Gy group, SAE1 siRNA group, and SAE1 siRNA + 6 Gy group at 24 h after radiation via flow cytometry (Figure 8A). The results showed irradiation with a dose of 6 Gy and transfection with SAE1 siRNA, both induced CRC apoptosis, and the apoptosis percentage of the SAE1 siRNA + 6 Gy group was higher than that of the other two groups in HCT116 (Figure 8B) and SW620 (Figure 8B) cells. To confirm these results, PARP and cleaved caspase-3 were detected using Western blotting. The results showed that cleaved PARP was found in the 6 Gy and SAE1 siRNA groups in HCT116 and SW620 cells. The protein expression of cleaved PARP in the SAE1 siRNA + 6 Gy group was higher than that in the other groups in HCT116 (Figure 8C) and SW620 cells (Figure 8D). However, cleaved caspase-3 was found in the 6 Gy as well as the SAE1 siRNA group in SW620 cells (Figure 8D), but not in HCT116 cells (Figure 8C). Cleaved caspase-3 protein expression in the SAE1 siRNA + 6 Gy group was higher than that in the other groups in HCT116 (Figure 8C) and SW620 cells (Figure 8D). These data support the hypothesis that SAE1 regulates the effects of radiotherapy in CRC.

## 4. Discussion

The clinicopathological parameters of colorectal cancer are important factors that influence the prognosis and treatment of the disease, such as tumor stage, histological grade, presence of lymph node metastasis, and tumor biomarkers [17]. The TNM system is used to stage colorectal cancer, which takes into account the size and depth of the tumor (T), whether it has spread to nearby lymph nodes (N), and whether it has metastasized to other organs (M) [18,19]. In addition, the location of the tumor within the colon or rectum can affect the symptoms, treatment options, and prognosis. Tumors in the right colon tend to cause more bleeding and anemia, while tumors in the left colon or rectum may cause changes in bowel habits, pain, and incomplete emptying. Some biomarkers, such as microsatellite instability (MSI) and the presence of certain genetic mutations, can help predict how well a person is likely to respond to treatment and may affect treatment decisions [20]. SUMOylation is a multi-step enzymatic cascade that regulates multiple biological functions, including tumor development [11]. In addition, the SUMOylation pathway, which includes the dimeric SUMO E1 SAE1/UBA2, single E2 Ubc9, and E3 ligases, regulates many cellular functions, such as cell growth, proliferation, apoptosis, DNA repair, and cell survival [21,22,23]. Emerging evidence has shown that abnormal SUMOylation may cause carcinogenesis, by influencing abnormal cell proliferation, apoptosis resistance, and metastatic potential [12]. SAE1 is essential for normal cellular functions and development. Mutations in the SAE1 gene have been linked to several diseases, including cancer, neurological disorders, and skeletal dysplasia [21,22,23].

Previous studies have shown that higher SAE1 and SAE2 expressions in patients with breast cancer are associated with significantly higher instances of metastasis and poor prognosis [24,25]. Furthermore, high SAE1 protein expression is known to have a strongly significant correlation with metastasis and disease progression in hepatocellular carcinoma [14,25]. It has been revealed that upregulated SAE1 is related with a higher grade of tumor malignancy and poor prognosis in glioma patients [16]. In cancer, SAE1 has been implicated in the regulation of tumor growth and metastasis, as well as the response to chemotherapy and radiation therapy. Some studies have suggested that inhibiting SAE1 activity may be a potential strategy for cancer treatment. Overall, SAE1 is an important protein that plays a critical role in the regulation of cellular processes and disease development, and it is an active area of research in various fields, including cancer biology and drug discovery [14,16,24,25].

In our study, SAE1 protein expression was significantly correlated with the M stage, and higher SAE1 expression was significantly correlated with poor overall survival. In addition, SAE1 was significantly associated with overall survival time in the multivariate analysis with Cox regression. These results suggest that SAE1 is an independent prognostic biomarker in colorectal cancer (CRC). Previous studies have shown that upregulated SAE1 promotes cell proliferation in hepatocellular carcinoma [14] and promotes cell progression in vitro and in vivo in gliomas [16]. Our results showed that knockdown of SAE1 attenuated CRC cell proliferation both in vitro and in vivo. In addition, silencing SAE1 by SAE1 siRNA increased cleaved PARP, a biomarker of apoptosis, and decreased cyclin D1, a biomarker of the cell cycle and protein expression. Therefore, it can be stated that SAE1 plays an important role in the regulation of tumor progression in CRC.

SUMOylation is known to regulate DNA repair following radiation-induced damage [26,27]. UV-radiation induces Rpb1 and DDB2 SUMOylation [28,29]. SUMO2-regulated SUMOylation of SH3GLB1 promotes ionizing radiosensitivity [30]. In addition, SUMOylation of PAF1/PD2 has been demonstrated to induce radio-resistance in pancreatic ductal adenocarcinoma [31]. However, the mechanism underlying the relationship between SAE1 and radiation remains unclear. In our study, SAE1 knockdown decreased colony formation and increased radiation-induced expression of cleaved PARP and cleaved caspase-3 at 6 Gy radiation. These data support the hypothesis that the silencing of SAE1 leads to enhanced radiosensitivity in CRC.

However, we must acknowledge several limitations in this study. Despite the relatively higher overall survival rate in colorectal cancer, the issue of recurrence remains significant. Regrettably, our study lacks statistical results pertaining to progression-free survival, which is crucial for a comprehensive assessment of treatment outcomes. Furthermore, the mechanistic exploration in our study might be considered limited in depth. While we have illuminated the potential of SAE-1 in colorectal cancer treatment, a more thorough investigation into the underlying mechanisms is warranted. This aspect stands as a direction for future research endeavors.

## 5. Conclusions

The results of this study established a correlation between high SAE1 expression and poor prognosis in colorectal cancer patients. In addition, SAE1 was identified as an independent biomarker of overall survival in patients with colorectal cancer. SAE1 knockdown inhibited cell proliferation in vitro and in vivo, decreased the protein expression of cyclin D1, increased PARP protein expression, and promoted radiosensitivity in colorectal cancer cells. These findings suggest that SAE1 may serve as a potential therapeutic target for the disease. Future research in this area should focus on targeting SAE1 expression via inhibitors or RNAi as a feasible treatment option for colorectal cancer.

## Figures and Tables

**Table 1:** SAE-1 expression correlated with clinicopathologic parameters in CRC.

	Number of Patients	SAE-1 Expression (*n*, %)		*p*-Value
		Low	High	
**Sex**				0.414
Male	100	61 (37.9%)	39 (24.2%)	
Female	61	33 (20.5%)	28 (17.4%)	
**Age**				0.187
≥60	37	18 (11.2%)	19 (11.8%)	
<60	124	76 (47.2%)	48 (29.8%)	
**Tumor size**				0.191
≥5 cm	60	31 (19.3%)	29 (18.0%)	
<5 cm	101	63 (39.1%)	17 (12.3%)	
**T stage**				1
T1 + T2	56	33 (20.5%)	23 (14.3%)	
T3 + T4	105	61 (37.9%)	44 (27.3%)	
**N stage**				0.053
N0	94	61 (37.9%)	33 (20.5%)	
N1 + 2	67	33 (20.5%)	34 (21.1%)	
**M stage**				0.006
M0	120	78 (48.4%)	42 (26.1%)	
M1	41	16 (9.9%)	25 (15.5%)	
**Pathologic stage**				0.081
I + II	83	54 (33.5%)	29 (18%)	
III + VI	78	40 (24.8%)	38 (23.6%)	
**Recurrent**				0.327
Yes	60	32 (19.9%)	28 (17.4%)	
No	101	62 (38.5%)	39 (24.2%)	
**Vascular invasion**				0.268
Yes	78	42 (26.1%)	26 (22.4%)	
No	83	52 (32.3%)	31 (19.3%)	
**Perineural invasion**				0.423
Yes	31	16 (9.9%)	15 (9.3%)	
No	130	78 (48.4%)	52 (32.3%)	

**Table 2:** Univariate and multivariate Cox regression analyses of overall survival in patients with CRC.

Overall Survival (OS)						
	Univariate Analysis			Multivariate Analysis		
	Relative Risk	95% CI	*p*-Value	Relative Risk	95% CI	*p*-Value
Sex	0.958	0.591–1.552	0.860			
Age	1.531	0.804–2.917	0.195			
Tumor size	0.984	0.605–1.599	0.948			
T stage	0.543	0.313–0.941	0.029	1.178	0.622–2.232	0.615
N stage	0.323	0.200–0.521	<0.001	0.888	0.313–2.520	0.823
M stage	0.100	0.059–0.170	<0.001	0.170	0.085–0.339	<0.001
Pathologic stage	0.215	0.128–0.361	<0.001	0.478	0.155–1.476	0.199
Recurrence	0.567	0.355–0.907	0.018	0.764	0.458–1.275	0.304
SAE-1	0.340	0.205–0.562	<0.001	0.383	0.223–0.655	<0.001
Vascular invasion	0.611	0.207–0.554	<0.001	0.937	0.314–2.799	0.908
Perineural invasion	0.042	0.336–0.947	0.03	1.110	0.618–1.992	0.728

CI, confidence interval.

## Data Availability

All data generated or analyzed during this study are included in this published article.
